# Calcium Dobesilate Ameliorates Cisplatin-induced Hepatotoxicity by Inhibiting Liver Oxidative Stress in Mice

**DOI:** 10.5812/ijpr-126613

**Published:** 2023-04-14

**Authors:** Gholamreza Bazmandegan, Zahra Kamiab, Amirmohammad Shafiei, Morteza Khademalhosseini, Ayat Kaeidi

**Affiliations:** 1 Physiology-Pharmacology Research Center, Research Institute of Basic Medical Sciences, Rafsanjan University of Medical Sciences, Rafsanjan, Iran; 2 Department of Physiology and Pharmacology, Rafsanjan University of Medical Sciences, Rafsanjan, Iran; 3 Department of Community Medicine, School of Medicine, Rafsanjan University of Medical Sciences, Rafsanjan, Iran; 4 Department of Pathology, School of Medicine, Rafsanjan University of Medical Sciences, Rafsanjan, Iran

**Keywords:** Calcium Dobesilate, Cisplatin, Oxidative Stress, Hepatotoxicity

## Abstract

**Background:**

Cisplatin has potent antitumor properties. It has several toxic side effects, such as hepatotoxicity. It is thought that hepatotoxicity induced by cisplatin is caused by oxidative stress.

**Objectives:**

It has shown that calcium dobesilate (CD) has potent antioxidant properties. The present study aimed to assess CD protective effects on cisplatin-induced hepatotoxicity in mice.

**Methods:**

In this study, 28 mice were selected randomly and were divided into four groups, including control, cisplatin (20 mg/kg, i.p., only on the first day of the experiment), Cisplatin+CD 50 (50 mg/kg CD, orally), and Cisplatin+CD 100 (cisplatin with 100 mg/kg CD, orally). A 4-day oral gavage of CD was applied to the treated groups. The mice were sacrificed on the 5th day, and serum glutamic pyruvic transaminase (SGPT), serum glutamic-oxaloacetic transaminase (SGOT), alkaline phosphatase (ALP), malondialdehyde (MDA) and reactive oxygen species (ROS) levels, superoxide dismutase (SOD), and glutathione peroxidase (GPx) enzyme activity levels in liver tissue were evaluated. Histopathological evaluation was assessed using hematoxylin and eosin-stained liver tissue sections.

**Results:**

The results indicated that there was a significant increase in GSPT, SGOT, ALP, and MDA and also a significant reduction in the liver activity of SOD and GPx in cisplatin-treated animals. Treatment with CD (100 mg/kg) remarkably attenuated the GSPT, SGOT, ALP, MDA, and ROS levels. Moreover, CD (100 mg/kg) elevated the SOD and GPx activity in the liver tissue of cisplatin-treated mice.

**Conclusions:**

The findings showed that CD has a protective effect against cisplatin-induced hepatotoxicity, at least by improving the antioxidant parameters.

## 1. Background

Cisplatin is an important cytotoxic agent with cellular alkylating action used in chemotherapy for various cancers, such as lung, ovary brain, and carcinoma (1). Despite cisplatin's potent antitumor properties, it has several toxic side effects, such as ototoxicity, neurotoxicity, nephrotoxicity, and hepatotoxicity, limiting its clinical use (2-4). Cisplatin-induced hepatotoxicity might be due to its metabolite accumulation in the liver (5). One of the most important mechanisms of cisplatin side effects is the overproduction of reactive oxygen species (ROS), causing oxidative stress, although these mechanisms are still unknown (6, 7). Cisplatin-induced oxidative stress results in tissue degradation, lipid peroxidation, protein and nucleic acid oxidation, and cell membrane degradation (8). It has been shown that these damages to liver tissue increase blood levels of liver enzymes, including alanine transaminase (ALT), alkaline phosphatase (ALP), and aspartate aminotransferase (AST) (9, 10). Moreover, the molecular changes consist of a decrease in antioxidant enzymes, such as glutathione peroxidase (GPx) and superoxide dismutase (SOD) in liver tissue, and also an increase in malondialdehyde (MDA) concentration (11, 12). Furthermore, histopathological changes, such as necrosis, inflammation, and vascular injuries, were found in the livers of cisplatin-treated animals (12, 13).

More than 40 years ago, calcium dobesilate (CD), one of cyclohexadiene bisulfate derivatives, was introduced to treat diabetic retinopathy due to its potential to decrease vascular permeability (14). Moreover, potent antioxidant properties have been confirmed in CD (15). In vitro studies have reported oxygen-free radical scavenging functions in this drug. Nevertheless, these experiences have also been confirmed using in vivo studies (16). In the study conducted by Jafarey et al., they reported that CD could decrease free ROS and increase the antioxidant enzymes, including GPx and SOD, in gentamicin-induced nephrotoxicity (17).

## 2. Objectives

Accordingly, it is necessary to find a drug that can reduce the hepatotoxicity of cisplatin. On the other hand, it seems that CD could have good protective effects under this condition. Thus, the current study aimed to assess whether CD affects cisplatin-induced liver damage in mice by studying histological alterations and oxidative stress indices.

## 3. Methods

### 3.1. Main Chemicals and Reagents

CD (Doxium®) was provided by OM PHARMA Co. (Switzerland), and cisplatin (CISPLATIN MYLAN®) was provided by MYLAN Co. (France).

### 3.2. Animals

In this study, 28 male mice were randomly selected from the animal house of Rafsanjan University of Medical Sciences, Rafsanjan, Iran. They were kept in polycarbonate cages with free access to food (Pars Industrial Co., Iran) and water under 24 ± 2°C room temperature with a 12-hour light/dark cycle. All animal experimental steps were performed according to the instructions for the care and use of laboratory animals at Rafsanjan University of Medical Sciences. The experimental procedures were approved by the local Ethical Committee (ethics code: IR.RUMS.REC.1397.028) and conducted in accordance with the standard ethical guidelines (NIH, publication no. 85-23, revised 1985; European Communities Directive 2010/63/EU).

### 3.3. Experimental Groups

In this experimental investigation, the mice were randomly divided into 4 groups (7 mice per group) as follows: (1) control (no particular treatment); (2) cisplatin (20 mg/kg, intraperitoneal; i.p.), on the first day of the study); and (3) and (4) cisplatin (20 mg/kg, on the first day of the study; i.p.) +CD (50 and 100 mg/kg/day oral administration, started from the 1st day and continued for 3 consecutive days) (18, 19).

### 3.4. Sample Collection

Twenty-four h after the last CD administration, diethyl ether was used to anesthetize the animals, and blood samples were collected through the cardiac puncture. Then, to collect blood serum, the samples were centrifuged at 1000 rpm for 3 minutes. Then, to measure the serum glutamic pyruvic transaminase (SGPT), serum glutamic-oxaloacetic transaminase (SGOT), and ALP levels, the serum samples were stored at -80 °C. The animals were sacrificed by decapitation, and their livers were harvested quickly. In order to conduct histopathological studies, half of the liver was fixed in a 10% formalin solution, and the other half was frozen in a nitrogen tank to assess the oxidative stress indices (18, 20).

### 3.5. Serum Parameters

An Auto Analyzer (Mindray, Guangzhou, China) with relevant commercial kits (ParsAzmoon Co., Tehran, Iran) was used to measure SGPT, SGOT, and ALP serum levels (20, 21).

### 3.6. Oxidative Parameters

The frozen livers were defrosted and homogenized using phosphate-buffered saline, and to collect the supernatants, they were centrifuged at 6000 rpm for 25 min at 4ºC (22, 23). The liver MDA concentration, SOD, and GPx enzyme activity levels were assessed in the collected supernatants using relevant kits (ZellBio, Veltlinerweg, Germany). The ROS level analysis was done with a chemiluminescence (CL) assay kit (Berthold Technologies, Germany) (24).

### 3.7. Histopathological Studies

Hematoxylin and eosin (H&E) were used to stain the prepared liver sections, and then they were observed under a light microscope in a blind manner (Nikon Labophot, Japan) with the help of an expert pathologist. The Ishak fibrosis score and the modified Knodell histology activity index (HAI) and Ishak fibrosis score were utilized to score the biopsy specimens (25, 26).

### 3.8. Statistical Analysis

The data were statistically analyzed with GraphPad Prism software (version 6, USA). The study results were presented as mean ± SEM. ANOVA, followed by Tukey’s post-hoc analysis, was used to determine the differences between the groups. A P-value less than 0.05 was considered statistically significant.

## 4. Results

### 4.1. Serum Parameters

**Figure 1.**
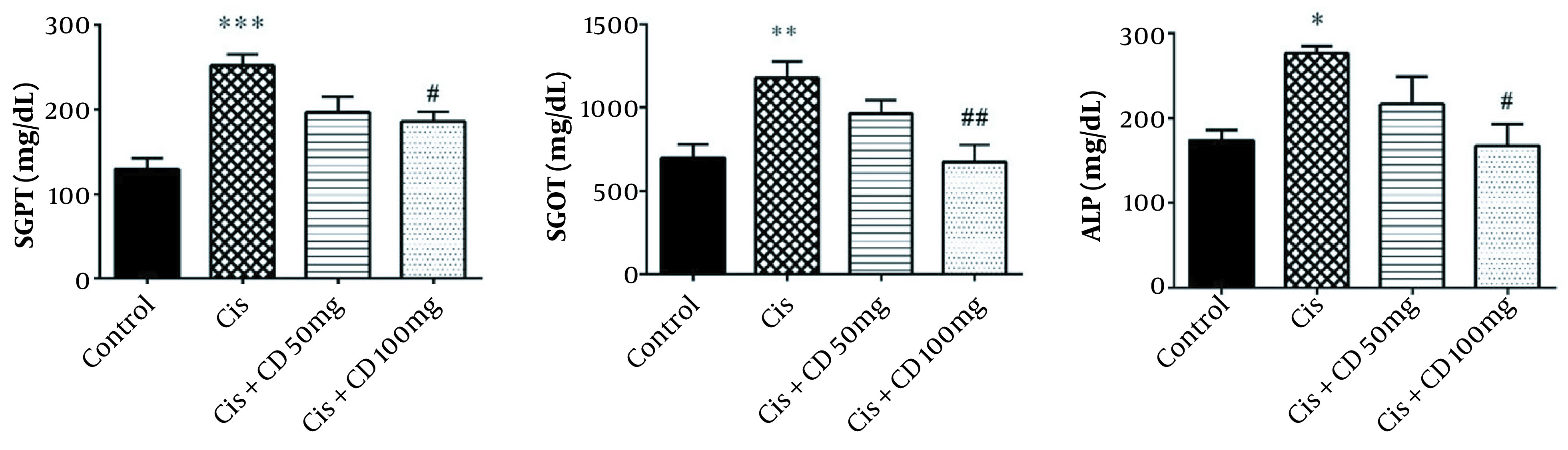
The data were represented as mean ± SEM. The effect of treatment with CD on SGPT (A), SGOT (B), and ALP (C) levels in cisplatin-induced hepatotoxicity. *P < 0.05, **P < 0.01 and ***P < 0.001: Compared to the control group. #P < 0.05 and ##P < 0.01: Compared to cisplatin group. CD: Calcium dobesilate.

The mean serum levels of SGPT, SGOT, and ALP in the cisplatin group significantly increased (P < 0.001, P < 0.01, and P < 0.05, respectively) (Figure 1). A significant reduction was observed in the SGPT, SGOT, and ALP serum levels by administrating CD at a dose of 100 mg/kg for 4 constitutive days compared to cisplatin group (P < 0.05, P < 0.01 and P < 0.05, respectively).

### 4.2. Oxidative Stress Assessments

**Figure 2.**
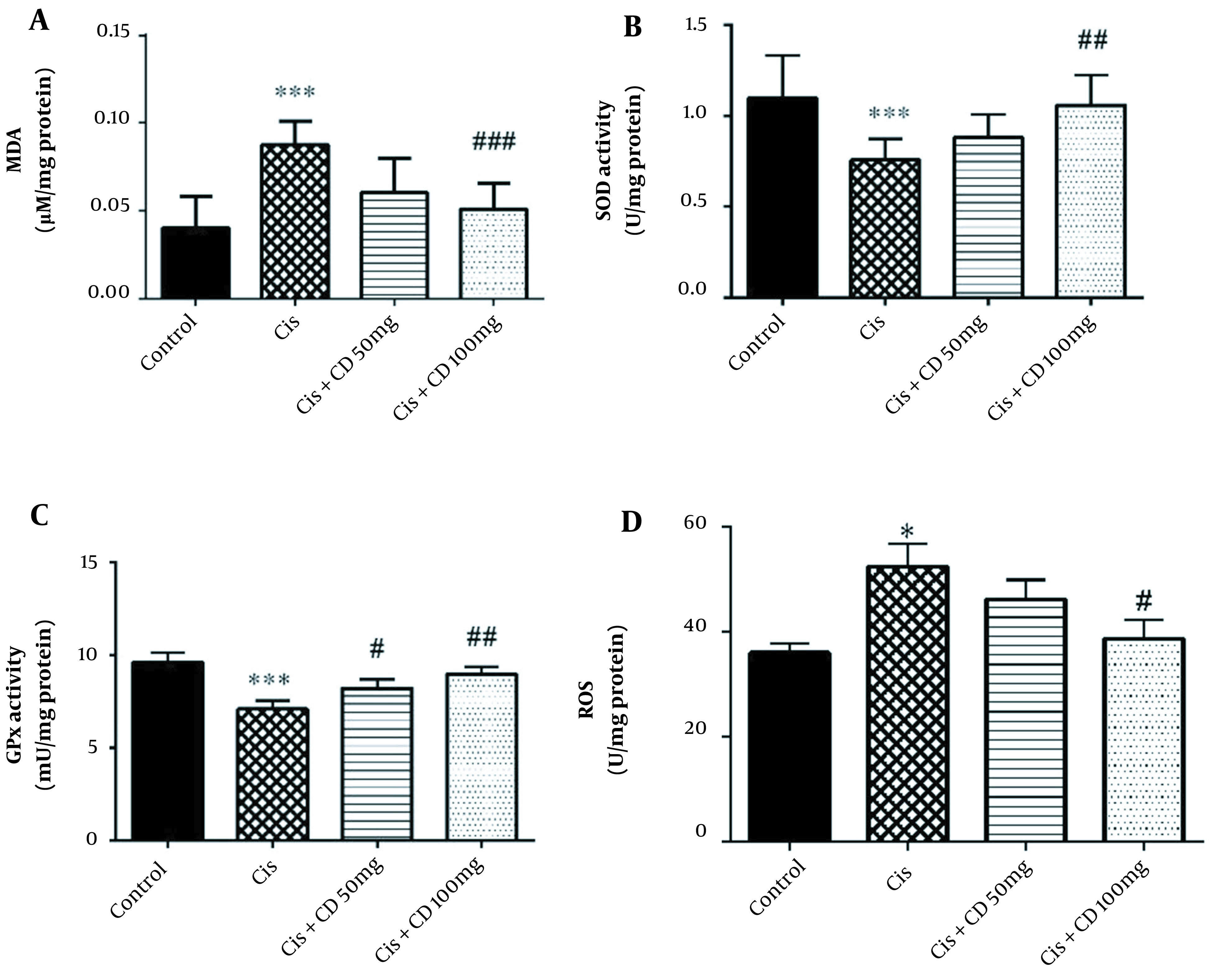
The data were represented as mean ± SEM. The treatment effect of CD on MDA (A), SOD (B), GPx (C), ROS (D) in cisplatin-induced hepatotoxicity. *P < 0.05 and ***P < 0.001: Compared to the control group. # P < 0.05, ## P < 0.01 and ###P < 0.001: Compared to cisplatin group.

The study results indicated that cisplatin administration significantly increased MDA concentration in comparison to the control group (P < 0.001) (Figure 2A). Moreover, a significant reduction in the mean concentration of MDA was observed in the group administrated 100 mg/kg CD for 4 constitutive days in comparison to cisplatin group (P < 0.001).

The study results demonstrated that the activities of SOD and GPx were significantly reduced by administrating cisplatin compared to the control group (P < 0.001) (Figure 2B and C). The SOD activity significantly increased by administrating CD (100 mg/kg) for 4 constitutive days compared to the cisplatin-treated animals (P < 0.01). Moreover, the GPx activity significantly increased by administrating CD at both doses of 50 and 100 mg/kg for 4 constitutive days compared to the cisplatin group (respectively, P < 0.05 and P < 0.01).

Our results also showed that cisplatin significantly increased the ROS concentration rather than the control group (P < 0.05) (Figure 2D). Furthermore, CD (100 mg/kg) could decrease the MDA concentration in Cisplatin + CD (100 mg/kg) versus the cisplatin group (P < 0.05).

### 4.3. The Effect of CD on Histopathological Indices in Cisplatin-induced Hepatotoxicity

**Figure 3.**
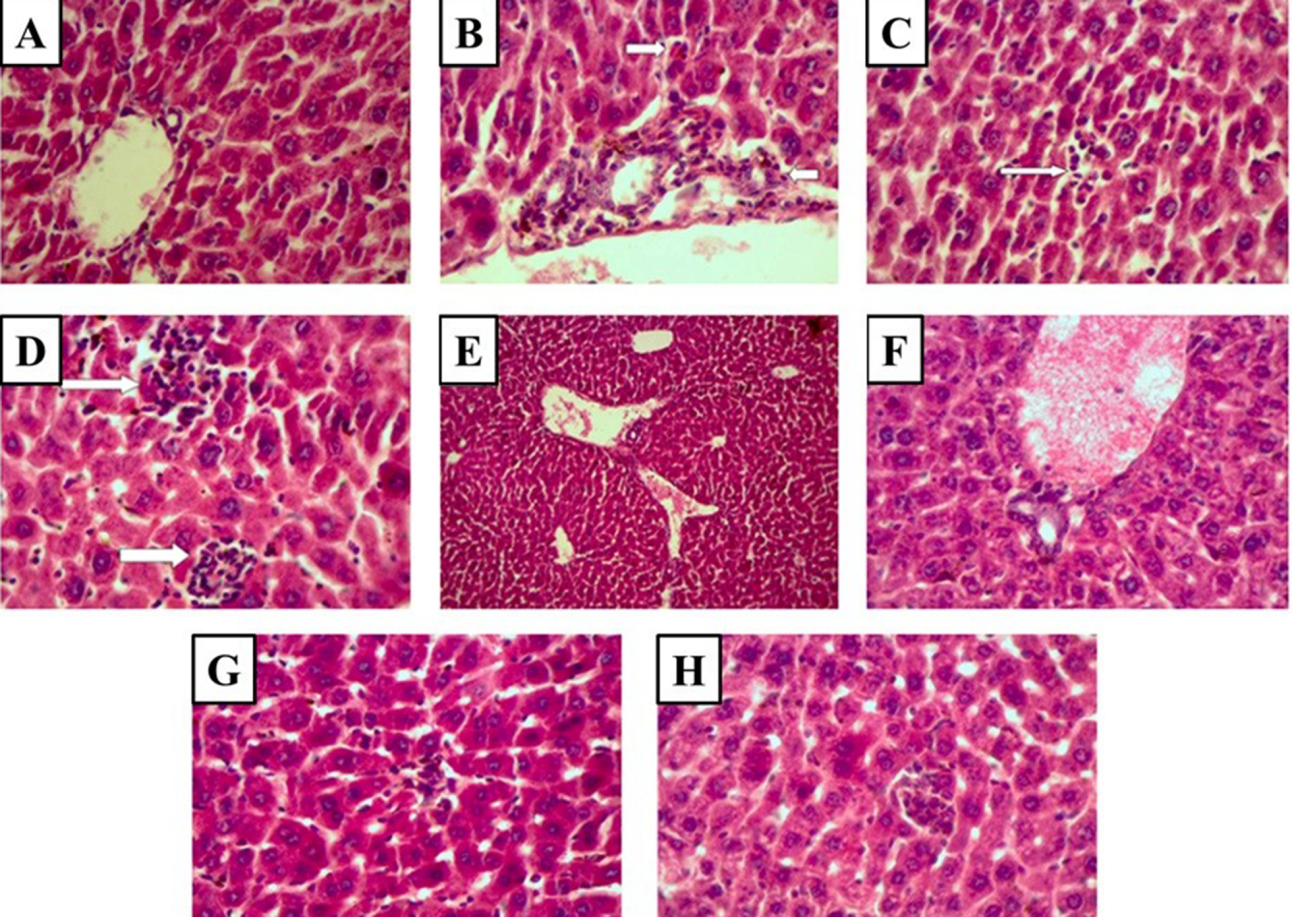
Histopathological observations (the stained liver sections using H&E; magnification X 400) reveal CD effects on cisplatin-induced hepatotoxicity changes in the liver. (A) Control (non-cisplatin treated). (B) Mild portal and periportal inflammation in some portal areas in the cisplatin group. (C) Focal lytic necrosis in the cisplatin group. (D) Confluent necrosis in the cisplatin group. (E) Portal inflammation in the cisplatin + 50 mg/kg CD group. (F) Portal inflammation in cisplatin + 100 mg/kg CD group. (G) Focal lytic necrosis in cisplatin + 50 mg/kg CD group. (H) Focal lytic necrosis in cisplatin + 100 mg/kg CD group.

No pathological lesions were seen in the liver of the control group (Figure 3A and Table 1). Extensive pathological lesions, such as periportal inflammation, focal lytic necrosis, and confluent necrosis due to cisplatin administration, were seen in the cisplatin-treated group (Figure 3B-D and Table 1). The cisplatin-induced pathological lesions dramatically decreased in the liver tissue by CD administration (100 and 500 mg/kg) (Figure 3E, F, G, H, and Table 1).

**Table 1.** The Effect of CD on Liver Histopathology in Cisplatin-induced Hepatotoxicity in Mice

	Portal Inflammation	Periportal Inflammation	Focal Lytic Necrosis	Confluent Necrosis	Grading Score
**Control**	0	0	0	0	0
**Cisplatin**	1	1	2	2	6
**Cisplatin + 50 mg/kg CD**	0	0	1	0	1
**Cisplatin + 100 mg/kg CD**	0	0	1	0	1

## 5. Discussion

Our results also revealed that oral administration of CD (50 and 100 mg/kg) for 4 constitutive days could effectively attenuate cisplatin's toxic effects on the liver tissue.

Although cisplatin is a strong antineoplastic drug, similar to other antineoplastic agents, it also has some serious adverse effects, such as hepatotoxicity, nephrotoxicity, and neurotoxicity (27, 28). It has been indicated that cisplatin-induced hepatotoxicity by elevated ROS generation, lipid peroxidation (oxidative stress), and histopathological lesions in the liver tissue (5). The incidence of these events in the liver leads to functional disorders characterized by increased SGPT, SGOT, and ALP levels (5, 13). In line with previous investigations, our data confirmed that cisplatin administration elevated the SGPT, SGOT, and ALP serum levels. Furthermore, the result of the present study showed that the treatment with 50 or 100 mg/kg CD reduced the SGPT, SGOT, and ALP increment levels in cisplatin-treated mice. Similar protective effects of antioxidant compounds have been reported in cisplatin-induced liver damage (9). It has been recently well documented that CD has strong antioxidant effects through increasing antioxidant enzyme activities and free radical scavenging (29, 30).

It has been found that cisplatin can elevate ROS generation in liver tissue by induction of oxidative stress (5, 31). Elevated cellular ROS production directly increases MDA generation by lipid peroxidation (32). Moreover, MDA has more potent reactive properties that have been enhanced in several studies after cisplatin administration (9, 11). Also, it seems that the over-production of ROS and MDA induces histopathological lesions and functional changes in liver tissue (33). Our data also showed that cisplatin administration reduces the SOD and GPx enzyme activities and increases the ROS and MDA (as cellular oxidative stress markers) concentration levels in liver tissue. In line with our study, it was established that treatment with CD significantly decreased MDA levels in liver tissue (34). Also, it has been demonstrated that CD oral administration (100 mg/kg/day) for 10 days can decrease oxidative stress indices such as MDA in the liver of experimental obstructive jaundice (35). Moreover, the CD could reduce MDA levels in isolated human varicose veins (36). Furthermore, CD attenuated the MDA levels in the retina of diabetic mice (37). Also, our previous study showed that CaD (100 mg/kg) could ameliorate oxidative stress by reducing the MDA concentration, as well as increasing the SOD and GPx activity levels) after CCL4-induced liver injury in mice. Therefore, it seems that CD increased its beneficial antioxidative effects by decreasing MDA.

According to previous studies (33, 38), cisplatin could decrease antioxidant enzyme levels (GPx, SOD). Accordingly, it was revealed that cisplatin reduced the antioxidant enzyme activities. Our findings also indicated that administering CD (mainly 100 mg/kg) to cisplatin-treated mice restored the activity of GPx and SOD. In line with our previous study, CD could decrease oxidative stress and support the antioxidant defense system, such as GPx and SOD (19). Also, in a human study, it was indicated that CD could improve the GPx and SOD levels and decrease the MDA level in cardiac surgery (39). Moreover, He and his colleagues showed that CD has protective effects on retinal damage in diabetic rats by elevating the GPx activity (40).

Several studies have demonstrated cisplatin-induced liver tissue damage (5, 13). In the present study, tissue lesions were found in the liver samples of cisplatin-treated animals. Also, our findings revealed that CD at doses of 50 and 100 mg/kg (more potentially) decreased these pathological lesions induced by cisplatin in the liver tissue. In this regard, Unal et al. found that CD reduced pathological liver damage in experimental obstructive jaundice in rats (35).

### 5.1. Conclusions

The findings of the present study showed that cisplatin caused oxidative stress and histopathological damage to rats' liver tissue. Moreover, CD administration (50 and 100 mg/kg) could reduce oxidative stress and histopathological changes in cisplatin-treated animals. Therefore, the findings indicated that CD could be used in the treatment of patients with cancer by decreasing cisplatin-induced liver damage.
